# The healing effect of bone marrow-derived stem cells in acute radiation syndrome

**DOI:** 10.12669/pjms.323.9895

**Published:** 2016

**Authors:** Seyed Mohammad Javad Mortazavi, Fatemeh Shekoohi-Shooli, Seyed Mahmood Reza Aghamir, Davood Mehrabani, Amirreza Dehghanian, Shahrokh Zare, Mohammad Amin Mosleh-Shirazi

**Affiliations:** 1 Seyed Mohammad Javad Mortazavi, Ionizing and Non-ionizing Radiation Protection Research Center, Shiraz University of Medical Sciences, Shiraz, Iran; 2 Fatemeh Shekoohi-Shooli^2^, Radiology and Radiotherapy Department, Shahid Beheshti University of Medical Science, Tehran, Iran; 3 Seyed Mahmood Reza Aghamir, Radiology and Radiotherapy Department, Shahid Beheshti University of Medical Science, Tehran, Iran; 4 Davood Mehrabani, Regenerative Medicine Department, University of Manitoba, Winnipeg, Manitoba, Canada. Stem Cell and Transgenic Technology Research Center, Shiraz University of Medical Sciences, Shiraz, Iran; 5 Amirreza Dehghanian, Trauma Research Center, Shahid Rajayee Hospital, Department of Pathology, School of Medicine, Shiraz University of Medical Sciences, Shiraz, Iran; 6 Shahrokh Zare, Stem Cell and Transgenic Technology Research Center, Shiraz University of Medical Sciences, Shiraz, Iran; 7 Mohammad Amin Mosleh-Shirazi, Ionizing and Non-ionizing Radiation Protection Research Center, Shiraz University of Medical Sciences, Shiraz, Iran

**Keywords:** Acute radiation syndrome, Mesenchymal stem cell, Bone marrow, Healing, Survival, Mouse

## Abstract

**Objectives:**

To determine the effect of bone marrow-derived mesenchymal stem cells (BMSCs) on regeneration of bone marrow and intestinal tissue and survival rate in experimental mice with acute radiation syndrome (ARS).

**Methods:**

Forty mice were randomly divided into two equal groups of A receiving no BMSC transplantation and B receiving BMSCs. BMSCs were isolated from the bone marrow and cultured in DMEM media. Both groups were irradiated with 10 Gy (dose rate 0.28 Gy/ min) ^60^CO during 35 minutes with a field size of 35×35 for all the body area. Twenty-four hours after γ irradiation, 150×10^3^ cells of passage 5 in 150 µl medium were injected intravenously into the tail. Animals were euthanized one and two weeks after cell transplantation. They were evaluated histologically for any changes in bone marrow and intestinal tissues. The survival rate in mice were also determined.

**Results:**

A significant increase for bone marrow cell count and survival rate were observed in group B in comparison to group A. Histological findings denoted to a healing in sample tissues.

**Conclusion:**

BMSCs could significantly reduce the side effects of ARS and increase the survival rate and healing in injured tissue. As such their transplantation may open a window in treatment of patients with ARS.

## INTRODUCTION

Acute radiation syndrome (ARS) is a problem that can be caused due to exposure by high lethal doses of ionizing radiation during a short period of time causing depletion of parenchymal cells in a tissue.^1^ High doses of ionizing radiation can cause detrimental systemic effects in organs such as the gastrointestinal tract, and blood circulation.^2^,^3^ So, patients afflicted with this syndrome must i) prevented and treated for any infection,^4^ ii) undergo hematopoiesis stimulation by administration of growth factors,^4^ iii) receive stem cells^4^,^5^ and iv) be protected physiologically from exposures.^4^

Stem cell transplantation was shown to be an effective approach that can be developed in the laboratory in severe radiation injuries.^6^ Mesenchymal stem cells (MSCs) are clonogenic undifferentiated cells with self-renewal and differentiation properties^7^ that have been isolated from tissues such as bone marrow,^8^ and adipose.^9^ They have multilineage properties and can be differentiated into osteoblasts.^10^

Today, MSCs are considered as an alternative treatment for functional recovery in patients and animals suffering from many diseases and disabilities.^11^ Bone marrow stem cells (BMSCs) have been used in several diseases such as ARS based on their safety and efficacy.^12^ The present study aimed to assess the effect of BMSCs on regeneration of bone marrow and intestinal tissue and survival rate in experimental mice with ARS.

## METHODS

BMSCs were isolated from male mice (weighing=27-30 g, age=8-12 weeks) that were provided from Laboratory Animal Center of Shiraz University of Medical Sciences. Animal housing and all experiments received the approval of the local animal care committee based on institutional guidelines and national animal welfare.

Animals were divided into 2 groups undergoing γ irradiation without any therapeutic intervention and the group undergoing γ irradiation with treatment intervention. Both groups were irradiated with 10 Gy (dose rate 0.28 Gy/ min) ^60^CO during 35 minutes with a field size of 35×35 for all the body area. Twenty-four hours after γ irradiation, 150×10^3^ cells of passage 5 in 150 µl medium were injected intravenously into the tail of treatment group. Animals were euthanized one and two weeks after cell transplantation for histological evaluation.

For MSC culture, the animals were euthanized and both femoral and tibial bones from each mouse were removed under sterile condition and after removal of muscular and connective tissues, the bones were cut at both ends and the bone marrow was flushed out into a 15 ml falcon tube using a 10 ml syringe filled with Dulbecco’s Modified Eagle Medium (DMEM; Biovet, Bulgaria) and 1% penicillin streptomycin (Sigma, USA). After isolation of bone marrow, they were transferred on ice and under sterile condition to stem cell laboratory (Stem Cell and Transgenic Technology Research Center, Shiraz University of Medical Sciences, Shiraz, Iran) for further follow up.

The bone marrow was diluted with an equal volume of DMEM, and centrifuged at 1200 rpm for 7 minutes and after removal of the supernatant, the precipitate was cultured in 25 cm^2^ flasks containing DMEM supplemented with 10% fetal bovine serum (FBS; Biovet, Bulgaria), 1% L-glutamine (Sigma, USA) and 1% penicillin and streptomycin. The culture flasks were transferred into CO2 incubator at 37°C with 5% CO2 and saturated humidity while the medium was changed after 24 and then every 3 days. The adherent cells were passaged at 80% confluency by washing with PBS twice (Gibco, USA) and then using 0.25% trypsin (Gibco, USA) for 3 min. The same volume of DMEM was added to inactivate the enzyme activity. Cell passaging was continued till passage 5.

The morphology of cells were assessed by inverted microscope (Olympus, USA). To enumerate the cells, BMSCs were plated in 24-well plates at a density of 5×10^4^ cell per well for one week. Three wells per day were evaluated for cell count and population doubling time (PDT). PDT was calculated using the formula PDT=T ln2/ln(Xe/Xb), while T was the incubation time in hours, Xb represented the cell number incubation starting time and Xe for the cell number at incubation ending time.

RT-PCR was conducted to evaluate the expression of MSC markers. In summary, after extraction of the total RNA using the column RNA isolation kit (Denazist-Asia, Iran based on manufacturer’s instructions, it was assessed by spectrophotometry. The complementary DNA (cDNA) was provided by AccuPower Cycle Script RT PreMix Kit (Bioneer, Korea) based on manufacturer’s guideline. For each reaction; 15 µL of total RNA was used to reach a volume of 20 µL with the DEPC water. Twelve thermal cycles was performed as follows: 30 sec at 20°C for primer annealing, 4 min at 42°C for cDNA synthesis, 30 sec at 55°C for melting secondary structure and cDNA synthesis and 5 minutes at 95°C for inactivation.

Then, 1 µL of template (cDNA) and PCR buffer, H2O, dNTPs, MgCl2, Taq DNA polymerase, and forward and reverse primers were mixed. The microtubules containing 20 µL of the mentioned mixture were placed into thermocycler (Eppendorf Mastercycler Gradient, Eppendorf, Hamburg, Germany) and 30 amplification cycles were undertaken (30 sec denaturation at 95°C, 30 sec annealing at 64°C, 62°C, and 61°C and 30 sec extension at 72°C with the 5 min at 95°C for primary denaturation and 5 min at 72°C for final extension). PCR products were assessed for defined bands by gel electrophoresis by DNA safe stain in 1.5% agarose gel medium. The bands were visualized using UV radiation by a gel documentation system (UVtec, Cambridge, UK) and photographed.

To determine the osteogenic differentiation, cells from passage 5 were seeded into 6 well plates. At 70-80% cell confluency, they were cultured for 21 days with low glucose DMEM containing 100 nM dexamethasone (Sigma, USA), 0.05l M ascorbate-2-phosphate (Wako Chemicals, USA), 10 mM b-glycerophosphate (Sigma, USA), 1% penicillin/streptomycin and 10% FBS. The medium was changed every 3 days. At the end of the third week, osteogenic differentiation was evaluated with Alizarin Red staining (Sigma, USA).

Both groups were evaluated histologically for any changes in bone marrow and intestinal tissues. For this purpose, formalin fixed paraffin embedded (FFPE) blocks were prepared from the femoral bone and small intestine. Hematoxylin and eosin (H&E) staining was used to evaluate the changes. Bone Marrow components were examined for bone marrow cellularity, necrosis, hemorrhage, fibrosis, myeloid to erythroid ratio and searched for regenerated foci. These findings were scored accordingly: (-) for absence of the change, (F) for focal, (+) for mild, (++) for moderate and (+++) for frank and widespread presence of the changes in subsequent examined tissue and these findings were compared between the two groups.

The results of the experiments are represented as the mean±standard deviation (SD). Data were analyzed using T-test for number of cells in the bone marrow and Kaplan-Meier analysis for survival experiments using SAS 9 software. Also, Mann-Whitney was used to evaluate the nonparametric data in small intestine tissue evaluating for presence (+) or absence (-) of necrosis, apoptotic changes and also goblet cell depletion.

## RESULTS

**Fig.1:**
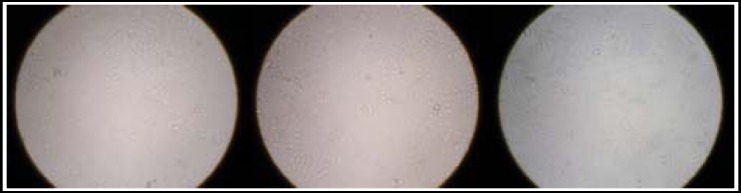
Bone marrow-derived mesenchymal stem cells in different passages. A: 1st, B: 2nd, and C: 3rd passage.

**Fig.2:**
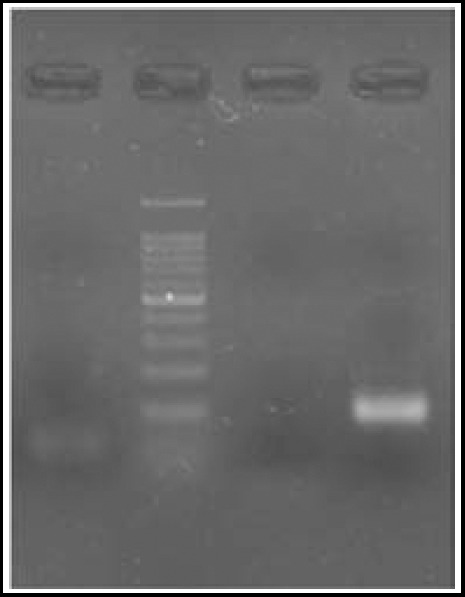
RT-PCR showed the presence of CD90 and lack of CD34 and CD45 (Ladder, CD90, CD34, 3: CD45).

**Fig.3:**
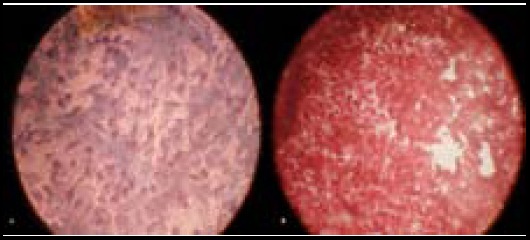
Alizarin red staining demonstrated calcification and osteogenic differentiation of BMSCs in mice. A: Control, B: Osteogenic differentiation.

**Fig.4:**
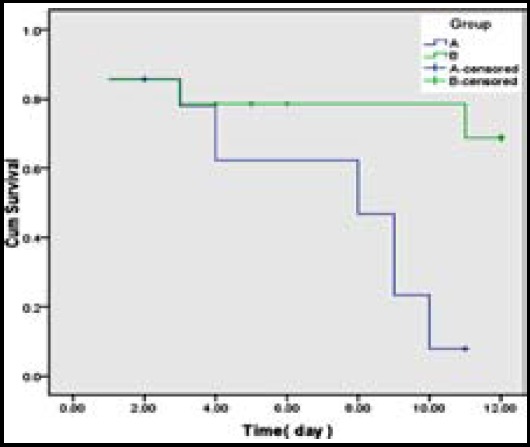
The effect of BMSCs injection on survival of mice exposed to 10 Gy irradiation to whole body.

BMSCs were plastic adherent and spindle-shape throughout all passages (Fig. 1A-1C). BMSCs expressed CD90 marker of mesenchymal stem cells but not CD34 and CD45 as markers for hematopoietic stem cells (Fig.2). Culture of BMSCs in osteogenic media lead to osteogenic differentiation of the BMSCs based on presence of calcium deposits after three weeks of staining with Alizarin red (Fig.3). A significant increase in survival rate was noticed two weeks after γ irradiation in group B (71.4%) when compared to group A (14.3%) (*p*=0.005). The survival rate estimated by Kaplan-Meier test showed that exposure of whole body to 10 Gy γ irradiation and then transplantation of BMSCs could significantly increase the survival rate in group B (Fig.4).

**Fig.5:**
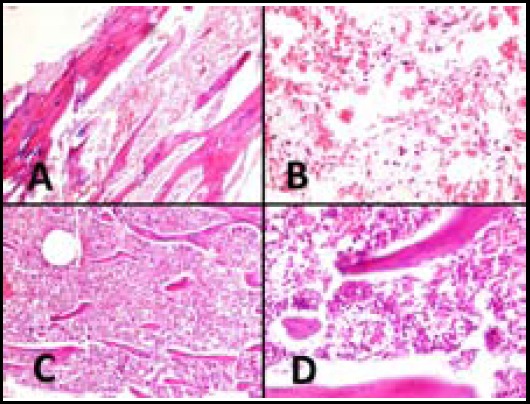
Histopathological changes in bone marrow of both groups. A (x100) and B (x400): Bone marrow shows diffuse hemorrhage, necrosis and loss of hematopoietic cells in Group A. C (X100) and D (X400): Bone marrow hyperceullarity and absence of bone marrow necrosis and hemorrhage.

**Fig.6:**
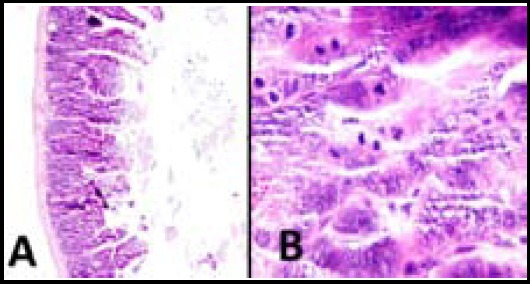
Histopathological changes of small intestine in group B. A (x100) and B (X400): Mucosal atrophy, increase in number of apoptosis and loss of goblet cells are seen.

Bone marrow necrosis, hemorrhage and hypocellularity were the main findings in the bone marrow in the Group A as compared with Group B (Table-I, Fig.5). These changes were statistically significant (*p*<0.05). For bone marrow fibrosis, the difference was not statistically significant (*p*>0.05). The myeloid to erythroid ratio was more than one in all of the cases of the group B. As seen in Table-II and Fig.6, small intestine of the two groups were evaluated for any histopathology changes including goblet cell depletion, necrosis and increase in number of apoptotic cells which were compared between two groups. There was a statistically significant increase in number of apoptotic cells in the Group A (*p*<0.05).

**Table-I:** A comparison between two groups regarding fibrosis, necrosis, hemorrhage and regeneration in the bone marrow.

*Group/Case*	*Fibrosis*	*Necrosis*	*Hemorrhage*	*Regeneration*
A				
1	+++	+	+	_
2	+	+++	++	_
3	+	+++	+++	F
4	+	+++	+++	_
5	_	+++	+++	F
6	_	++	+++	F
B				
1	_	_	_	_
2	_	_	_	_
3	_	_	_	_
4	_	_	_	_
5	_	F	_	_
6	_	_	_	_

**Table-II:** Comparison of two groups regarding apoptotic cells, necrosis and goblet cell depletion in the small intestine.

*Group/Case*	*Apoptosis*	*Necrosis*	*Goblet cell depletion*
A			
1	+	+	_
2	_	+	F
3	+	+	+
4	+	+	+
5	+	+	+
6	_	+	_
B			
1	_	+	+
2	+	+	+
3	_	+	+
4	_	+	+
5	+	+	_
6	+	+	+

## DISCUSSION

BMSCs are spindle shape similar to mesenchymal stem cells that have been confirmed in our findings too.^13^ We showed BMSCs could differentiate into osteoblast lineage secreting mineral matrix as one of the major components of bone matrix (calcium phosphate) detected by Alizarin red S staining as described before.^8^,^13^

The criteria of the Mesenchymal and Tissue Stem Cell Committee of the International Society for Cellular Therapy reported that stem cells that expressing the markers of CD44, CD90, CD73 and CD105 are considered mesenchymal and those that express markers of CD19, CD14, CD34 or CD45 are regarded hematopoietic stem cells.^14^ Our findings revealed positive marker of CD90 and lack of CD34 and CD45 markers at all passages by RT- PCR supporting the mesenchymal nature of the cells.

Comparison of the findings for both groups revealed that injection of BMSCs could significantly increase the number of cells in the bone marrow and the survival rate animals. Regeneration in bone marrow and healing in intestinal tissue were noticed in group B receiving BMSC transplantation in comparison to group A. These findings denoted to a reduction in the detrimental effects of ARS in mice. It was shown that BMSCs by increasing the secretion of growth factors, have the ability to differentiate into various cells and induce an immunomodulatory property through their paracrine and endocrine mechanisms leading to repair in injured tissues^9^ confirming the findings of the present study.

It was shown that transplantation of MSCs can be effective in treatment of ARS and reduce inflammation in tissue injuries.^12^ Also, in a 32-year-old man who was exposed to a dose of 14.5 GY γ irradiations in the whole body in an irradiation accident in China, it was found that cell therapy with MSCs was an effective therapeutic approach.^15^ Another study indicated that systemic administration of MSCs can stimulate mechanism counteracts the inflammatory events and also manage the detrimental effects of ARS after γ irradiation.^16^

Hu et al. reported that MSC transplantation would result into an increase in hematopoiesis and reduction of apoptosis, and it was suggested as a valuable strategy for management of ARS.^17^ MSCs were also shown to migrate and settle at the site of injury in the small intestine and lead to a healing and recovery for the existing injuries.^18^ Some authors reported therapeutic use of MSCs in compact bones to reduce the lesions and increase the survival rate after exposure to lethal doses of γ irradiation in the whole body.^6^

All the above-mentioned studies confirmed our findings in the present study. So, we showed that transplantation of BMSCs has detrimental effects in reduction of lesions in several tissues. BMSCs were demonstrated to increase the survival rate in ARS have healing effects in injured tissues that may be due to immunomudulating and anti-inflammatory effects of MSCs and an increase in secretion of growth factors and cytokines by these cells after γ irradiation.^19^

## CONCLUSION

Based on our findings, BMSCs were shown to reduce the detrimental effects of ARS and increase the survival rate in exposures to γ irradiation that can open a window in treatment of patients with ARS.

## References

[1] Kazzi Z, Buzzell J, Bertelli L, Christensen D (2015). Emergency department management of patients internally contaminated with radioactive material. Emerg Med Clin North Am.

[2] Dörr H, Meineke V (2011). Acute radiation syndrome caused by accidental radiation exposure - therapeutic principles. BMC Med.

[3] Gaberman E, Pinzur L, Levdansky L, Tsirlin M, Netzer N, Aberman Z (2013). Mitigation of lethal radiation syndrome in mice by intramuscular injection of 3D cultured adherent human placental stromal cells. PloS One.

[4] Becker SM (2011). Risk communication and radiological/nuclear terrorism:a strategic view. Health Phys.

[5] Gan J, Meng F, Zhou X, Li C, He Y, Zeng X (2015). Hematopoietic recovery of acute radiation syndrome by human superoxide dismutase–expressing umbilical cord mesenchymal stromal cells. Cytotherapy.

[6] Shukai Q, Hanyun R, Yongjin S, Wei L (2014). Allogeneic compact bone-derived mesenchymal stem cell transplantation increases survival of mice exposed to lethal total body irradiation:a potential immunological mechanism. Chin Med J. (Engl).

[7] Mehrabani D, Hassanshahi MA, Tamadon A, Zare S, Keshavarz S, Rahmanifar F (2015). Adipose tissue-derived mesenchymal stem cells repair germinal cells of seminiferous tubules of busulfan-induced azoospermic rats. J Hum Reprod Sci.

[8] Shaterzadeh Yazdi H, Mehrabani D, Khodakaram Tafti A, Dianatpour M, Zare S, Tamaddon A (2015). Osteogenic potential of subcutaneous adipose-derived stem cells in a rabbit model. Onl J Vet Res.

[9] Mehrabani D, Mehrabani G, Zare S, Manafi A (2013). Adipose-derived stem cells (ADSC) and aesthetic surgery:a mini review. World J Plast Surg.

[10] Shaterzadeh Yazdi H, Mehrabani D, Khodakaram Tafti A, Dianatpour M, Zare S, Tamaddon A (2015). Osteogenic potential of subcutaneous adipose-derived stem cells in a rabbit model. Onl J Vet Res.

[11] Ghobadi F, Mehrabani D, Mehrabani G (2015). Regenerative Potential of Endometrial Stem Cells:A Mini Review. World J Plast Surg.

[12] Eaton EB, Varney TR (2015). Mesenchymal stem cell therapy for acute radiation syndrome:innovative medical approaches in military medicine. Mil Med Res.

[13] Aliborzi G, Vahdati A, Hossini SE, Mehrabani D (2015). Evaluation of bone marrow-derived mesenchymal stem cells from Guinea pigs. Onl J Vet Res.

[14] Dominici M, Le Blanc K, Mueller I, Slaper-Cortenbach I, Marini F, Krause D (2006). Minimal criteria for defining multipotent mesenchymal stromal cells. The International Society for Cellular Therapy position statement. Cytotherapy.

[15] Guo M, Dong Z, Qiao J, Yu C, Sun Q, Hu K (2014). Severe acute radiation syndrome:treatment of a lethally 60Co-source irradiated accident victim in China with HLA-mismatched peripheral blood stem cell transplantation and mesenchymal stem cells. J Radiat Res.

[16] Lange C, Brunswig-Spickenheier B, Cappallo-Obermann H, Eggert K, Gehling UM, Rudolph C (2011). Radiation rescue:mesenchymal stromal cells protect from lethal irradiation. PloS One.

[17] Hu K, Sun Q, Guo M, Ai H (2010). The radiation protection and therapy effects of mesenchymal stem cells in mice with acute radiation injury. Br J Radiol.

[18] Chapel A, Bertho JM, Bensidhoum M, Fouillard L, Young RG, Frick J (2003). Mesenchyma stem cells home to injured tissues when co-infused with hematopoietic cells to treat a radiation-induced multi-organ failure syndrome. J Gene Med.

[19] Lee TK, Stupans I (2002). Radioprotection:the non-steroidal anti-inflammatory drugs (NSAIDs) and prostaglandins. J Pharm Pharmacol.

